# Persistent active avoidance correlates with activity in prelimbic cortex and ventral striatum

**DOI:** 10.3389/fnbeh.2015.00184

**Published:** 2015-07-15

**Authors:** Christian Bravo-Rivera, Ciorana Roman-Ortiz, Marlian Montesinos-Cartagena, Gregory J. Quirk

**Affiliations:** Departments of Psychiatry and Anatomy and Neurobiology, University of Puerto Rico School of MedicineSan Juan, Puerto Rico

**Keywords:** infralimbic, fear extinction, amygdala, c-Fos, freezing

## Abstract

Persistent avoidance is a prominent symptom of anxiety disorders and is often resistant to extinction-based therapies. Little is known about the circuitry mediating persistent avoidance. Using a recently described platform-mediated active avoidance task, we assessed activity in several structures with c-Fos immuno-labeling. In Task 1, rats were conditioned to avoid a tone-signaled shock by moving to a safe platform, and then were extinguished over two days. One day later, failure to retrieve extinction correlated with increased activity in the prelimbic prefrontal cortex (PL), ventral striatum (VS), and basal amygdala (BA), and decreased activity in infralimbic prefrontal cortex (IL), consistent with pharmacological inactivation studies. In Task 2, the platform was removed during extinction training and fear (suppression of bar pressing) was extinguished to criterion over 3–5 days. The platform was then returned in a post-extinction test. Under these conditions, avoidance levels were equivalent to Experiment 1 and correlated with increased activity in PL and VS, but there was no correlation with activity in IL or BA. Thus, persistent avoidance can occur independently of deficits in fear extinction and its associated structures.

## Introduction

To ensure survival, individuals must learn to actively avoid cues predictive of danger. Active avoidance can be extinguished when the cues no longer predict danger. A long standing theory proposes that avoidance is initially reinforced by fear, and it is subsequently reinforced by fear reduction (Mowrer and Lamoreaux, 1946). However, others studies have dissociated fear from avoidance (Lolordo and Rescorla, 1966; Riccio and Silvestri, 1973; Overmier and Brackbill, 1977). The majority of studies focus on neural mechanisms of avoidance acquisition (Gabriel et al., 1983; Orona and Gabriel, 1983; Gabriel, 1990; Maren et al., 1991; Savonenko et al., 1999; Holahan and White, 2002; Lázaro-Muñoz et al., 2010; Shumake et al., 2010; Darvas et al., 2011; Moscarello and LeDoux, 2013; Beck et al., 2014; Lichtenberg et al., 2014; Ramirez et al., 2015), but few focus on its extinction (Gabriel et al., 1983; Pang et al., 2010; Jiao et al., 2011; Bravo-Rivera et al., 2014b; Wendler et al., 2014). In a platform-mediated avoidance task (Bravo-Rivera et al., 2014b), rats learn to avoid a tone-signaled footshock by stepping onto a nearby platform at the expense of sucrose pellets. Pharmacological inactivation of prelimbic prefrontal cortex (PL) or ventral striatum (VS), but not infralimbic cortex (IL), impaired avoidance expression, whereas inactivation of the IL prior to avoidance extinction impaired retrieval of extinction the following day.

In anxiety patients, persistent avoidance is maladaptive and can severely impair quality of life (Aupperle et al., 2012). A percentage of rats persist in platform-mediated avoidance, despite extinction training (Bravo-Rivera et al., 2014b). We therefore sought to identify structures involved in persistent avoidance using c-Fos immuno-labeling. We focused on structures previously linked to avoidance expression, such as PL (Beck et al., 2014; Bravo-Rivera et al., 2014b) and VS (Darvas et al., 2011; Bravo-Rivera et al., 2014b; Ramirez et al., 2015), as well as structures linked to conditioned fear and fear extinction such as basal amygdala (BA; Herry et al., 2006; Laurent et al., 2008; Sierra-Mercado et al., 2011), and IL (Milad and Quirk, 2002; Sierra-Mercado et al., 2011; Do-Monte et al., 2015a), respectively. We used a correlational analysis to reveal individual variation across rats. Extinction of signaled avoidance has two components: (1) extinction of the tone-shock association (Rescorla and Heth, 1975); and (2) extinction of avoidance responding (Pang et al., 2010; Beck et al., 2011; Todd et al., 2014; Wendler et al., 2014). We therefore modified our extinction task to dissociate these two components.

## Materials and Methods

### Bar–Press Training

A total of 47 male Sprague–Dawley rats (Harlan Laboratories, Indianapolis, IN, USA) weighing 300–360 g were used in this study. Rats were restricted to 18 g/day of standard laboratory chow, followed by 10 days of training to press a bar for sucrose pellets on a variable interval schedule of reinforcement averaging 30 s (VI–30 s). Rats were trained until they reached a criterion of >10 presses/min. All procedures were approved by the Institutional Animal Care and Use Committee of the University of Puerto Rico School of Medicine, in compliance with National Institutes of Health’s Guide for the Care and Use of Laboratory Animals (Eighth Edition).

### Platform–Mediated Avoidance Training

We used the same parameters for the platform-mediated avoidance task as in our previous study (Bravo-Rivera et al., 2014b). Rats were trained in standard operant chambers (26.7 cm long, 27.9 cm wide, 27.9 cm tall; Coulbourn Instruments, Allentown, PA, USA) located in sound–attenuating cubicles (Med Associates, Burlington, VT, USA). The floor of the chambers consisted of stainless steel bars delivering a scrambled electric foot–shock. Shock grids and floor trays were cleaned with soap and water, and chamber walls were cleaned with wet paper towels. Sucrose pellets were available on a VI-30 s schedule throughout all phases of training and tests. Rats were conditioned with a pure tone (30 s, 4 kHz, 75 dB) co-terminating with a shock delivered through the floor grids (2 s, 0.4 mA). The inter–trial interval was variable, averaging 3 min. An acrylic square platform (14.0 cm each side, 0.33 cm tall) located in the opposite corner of the sucrose–delivering bar protected rats from the shock. The platform was fixed to the floor and was present during bar-press training prior to conditioning to reduce novelty. Rats were trained in platform-mediated avoidance for 10 days to reduce freezing and return spontaneous press rates to pre-conditioning levels. Each day, rats received three sessions consisting of three tone-shock trials each (9 tone–shock pairings per day). Rats were left in the training chamber between sessions for 5 min to reinforce bar–press training and to reduce contextual fear.

### Extinction Training

For Task 1, rats were presented daily with 15 unreinforced tones in the same conditioning chamber with the platform present. After two extinction training days, rats were presented with an avoidance test (two tones). For Task 2, the platform was removed prior to extinction, and rats were presented daily with 15 unreinforced tones. Given that freezing decreases to low levels with platform-mediated avoidance training, we used bar-press suppression as an index of fear memory (Bouton and Bolles, 1980; Quirk et al., 2000). A criterion of <25% suppression during the first two extinction trials was used to ensure adequate extinction, and rats were given 3–5 days of extinction training (without platform) to reach criterion. Two out of 24 rats were excluded from the experiment for failing to reach this criterion. One day following the conclusion of extinction training, the platform was returned and rats were presented with an avoidance test (two tones). Task 1 was designed such that it would reveal failure rats with a fixed amount of training that typically results in successful extinction, whereas Task 2 extinguished rats to criterion to normalize Pavlovian extinction such that it could not be a contributing factor to persistence. After the avoidance test in each task, a subset of rats exhibiting a wide range of avoidance values was selected for c-Fos immuno-labeling.

### Data Analysis

Behavior was recorded with digital video cameras (Micro Video Products, Bobcaygeon, ON, Canada) and freezing was automatically analyzed from video images (Freezescan, Clever Systems, Reston, VA, USA). The amount of time freezing to the tone was expressed as a percentage of the tone presentation (% Freezing). Avoidance was defined as the time spent with at least two paws on the platform, and was expressed as a percentage of the tone presentation (% Time on platform). We scored two paws on platform as avoidance because rats typically test the bars for shock with the two front paws during tone presentations. Moreover, rats cannot reach the sucrose–delivering bar from the platform, which constitutes a behavioral cost of avoidance. Avoidance was scored from videos by a trained observer. We also measured the percent of bar-press suppression to the tone (Bouton and Bolles, 1980; Quirk et al., 2000), calculated as follows: (pretone rate − tone rate)/(pretone rate + tone rate)*(100). A value of zero percent indicates no suppression, whereas a value of 100% indicates complete suppression. We performed regression analyses with the corresponding *F*-test and Student’s *t*-test (Statistica, StatSoft, Tulsa, OK, USA) throughout the study.

### Immunohistochemistry

We used a c-Fos immuno-labeling protocol that we previously described (Padilla-Coreano et al., 2012). One hour after the end of the behavioral test, rats were anesthetized with sodium pentobarbital (450 mg/Kg, i.p.) and then perfused transcardially with 250 ml of 0.9% saline followed by 500 ml of cold 4% paraformaldehyde (PFA) in 0.1 M phosphate buffer saline (PBS) at pH 7.4. Brains were removed and fixed overnight in 4% PFA, and transferred to 30% sucrose in 0.1 M PBS for 48 h, for cryoprotection. Frozen sections were cut coronally (40 μm) with a cryostat (CM 1850; Leica) at different levels of the prefrontal cortex, striatum, and amygdala.

All sections were washed in 0.1 M PBS with 0.1% tween (Tween-20, Sigma Aldrich, USA) between reactions three times for 5 min each. Sections were initially blocked in a solution of 2% normal goat serum (NGS, Vector Laboratories, Burlingame, CA, USA) and 0.1% tween in 0.1 M PBS (pH 7.4) for 1 h. Afterwards, sections were incubated overnight at room temperature with anti-c-Fos serum raised in rabbit (Ab-5, Oncogene Science, USA) at a concentration of 1:20,000. Sections were then incubated for 2 h at room temperature in a solution of biotinylated goat anti-rabbit IgG (Vector Laboratories, USA) and placed in a mixed avidin biotin horseradish peroxidase complex solution (ABC Elite Kit, Vector Laboratories, USA) for 90 min. Black/brown immuno-labeled nuclei labeled for c-Fos were visualized after 15 min of exposure to a solution containing 0.02% diaminobenzidine tetrahydrochloride with 0.3% nickel ammonium sulfate in 0.05 M Tris buffer, pH 7.6, followed by a 10 min incubation period in a chromogen solution with glucose oxidase (10%) and D-Glucose (10%). The reaction was stopped using three 5 min washes of 0.1 M PBS without tween. Sections were mounted on gelatin coated slides, dehydrated, and cover-slipped. Counter sections were collected, stained for Nissl bodies, cover-slipped, and used to determine the anatomical boundaries of each structure analyzed.

c-Fos immuno-labeled neurons were automatically counted at 20× magnification with an Olympus microscope (Model B×51) equipped with a digital camera. Micrographs were generated for prelimbic cortex (PL, +3.00 to +3.70 AP), infralimbic cortex (IL, +3.00 to +3.70 AP), orbitofrontal cortex (OFC, +3.00 to +3.70 AP), ventral striatum (VS, +2.00 to 0.00 AP), basolateral nucleus of the amygdala (BLA, −3.00 to −2.00 AP) and central nucleus of the amygdala, divided into lateral (CeL, −3.00 to −2.00 AP) and medial (CeM, −3.00 to −2.00 AP) portions, and paraventricular thalamic nucleus (PVT, −3.00 to −2.00 AP). Example of micrographs are shown in Figure 3B. The c-Fos immuno-labeled neuron counts were averaged for each hemisphere in 2–3 different sections for each structure (Metamorph software version 6.1). Density was calculated by dividing the number of c-Fos positive neurons by the total area of each region.

## Results

### Task 1: Extinction with Platform Present

**Figure 1:**
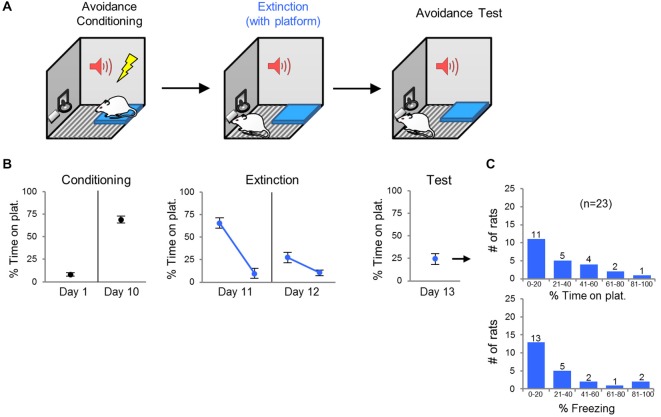
**Task 1: extinction with platform present. (A)** Rats were conditioned in platform mediated avoidance for 10 days, and then extinguished (15 unreinforced tones) for 2 days with the platform present throughout. One day following extinction training, rats received an avoidance test (two extinction trials). **(B)** Rats expressed high levels of avoidance by the end of conditioning. Avoidance expression was reduced following extinction training (days 11 and 12) and test (day 13). **(C)** Frequency histograms show the distribution of avoidance values (top) and freezing values (bottom) across the 23 rats. Error bars depict SEM.

Rats were given 10 days of avoidance conditioning, followed by 2 days of extinction training (tones without shocks; Figure 1A). On the first day of extinction (Day 11), the percent of time spent on the platform during the tone dropped from 66% to 10% (Figure 1B). The following day (day 12), rats started the session with relatively low levels of avoidance (27% of time on platform), indicating good retrieval of extinction (Figure 1B). During the avoidance test (day 13), rats showed minimal avoidance expression on average (24% of time on platform), again indicating retrieval of extinction (Figure 1B). However, 30% (*n* = 7) of the rats spent >40% of the time on the platform, indicating persistence of avoidance (Figure 1C); however, freezing and avoidance were not significantly correlated (*r* = 0.29, *p* = 0.17).

### Task 2: Extinction with Platform Absent

**Figure 2:**
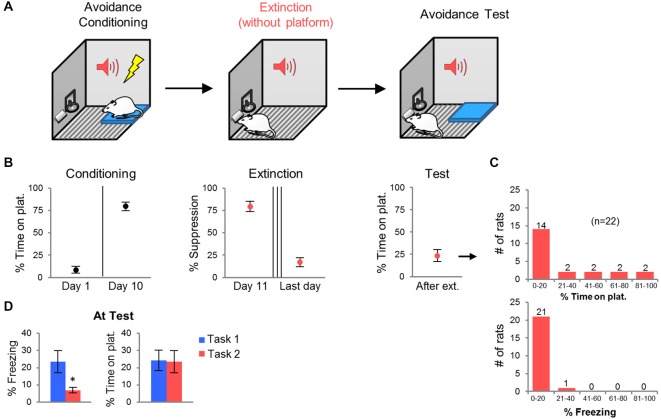
**Task 2: Extinction with platform absent. (A)** Rats were conditioned in platform-mediated avoidance (as in Task 1), but were extinguished with the platform absent. All rats were extinguished to the criterion of <25% suppression to the tone (3–5 days). The next day, following the last extinction session, the platform was returned for an avoidance test. **(B)** Conditioning increased avoidance whereas extinction decreased suppression. At test, the average avoidance level was reduced. **(C)** Frequency histograms show the distribution of avoidance values (top) and freezing values (bottom) across the 22 rats. **(D)** Compared to rats in Task 1, rats in Task 2 showed reduced freezing at test, but similar levels of avoidance. *p < 0.05.

Persistent avoidance could be due to an inability of rats to extinguish the tone-shock association across 2 days. Alternatively, persistent rats may be incapable of suppressing avoidance despite adequate extinction of the tone-shock association. In order to distinguish between these two possibilities, we modified the task by removing the platform during extinction training, and fully extinguishing the tone-shock association to criterion (Figure 2A). Given that freezing decreases to low levels with platform-mediated avoidance training, we used bar-press suppression as an index of tone-shock association memory (Bouton and Bolles, 1980; Quirk et al., 2000). Extinction sessions were repeated daily until each rat exhibited <25% suppression at the start of the session. Forty-five percent of rats reached this criterion by the 3^rd^ extinction session, 23% of rats required a 4^th^ session, and 32% required a 5^th^ session. One day following the last extinction session, the platform was returned and rats were tested for avoidance. We reasoned that persistent avoidance should be reduced if impairment in tone-shock extinction was the main factor driving persistence.

Extinction training reduced suppression of bar-pressing from 80 to 24% by the last day of extinction. Surprisingly, however, persistent avoidance still occurred at test (Figure 2C). The percentage of rats spending >40% of the time on the platform was similar to Task 1 (27% vs. 30% of rats). The average avoidance values for the group were also similar (Task 1: 24.2%, Task 2: 23.6%; Figure 2D). Freezing at test, however, was significantly lower in Task 2 compared to Task 1 (Task 1: 26.0%, Task 2: 6.9%, *t*_44_ = 2.40, *p* = 0.020; Figure 2D), despite equivalent freezing levels prior to extinction training (Task 1: 11.9%, Task 2: 14.3%, *t*_44_ = 0.31, *p* = 0.75). Thus, persistent avoidance can occur despite successful tone-shock extinction (Figure 2B). Next, we used c-Fos immuno-labeling to identify structures in which activity correlated with persistent avoidance.

### c-Fos Expression at Test in Task 1

**Figure 3:**
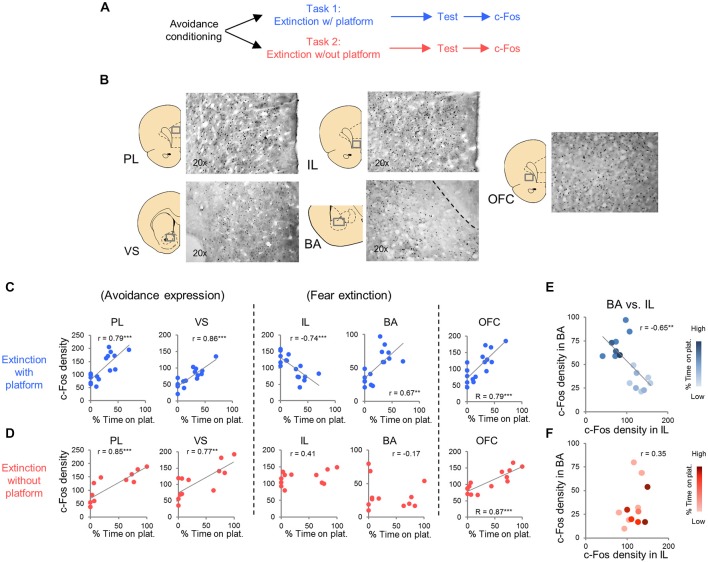
**c-Fos expression at post-extinction avoidance test. (A)** Schematic showing experimental protocol. **(B)** Representative micrographs of c-Fos immuno-labeling. **(C)** Task 1 revealed positive correlations between avoidance expression at test and c-Fos density in PL, ventral striatum (VS), basal amygdala (BA), and OFC, and a negative correlation in IL (correlation value in inset). **(D)** Task 2 revealed positive correlations in PL, VS and OFC only. **(E)** Comparison of c-Fos density in BA and IL in Task 1. **(F)** Comparison of c-Fos density in BA and IL in Task 2. Darker hues represent higher avoidance whereas lighter hues represent lower avoidance. ***p < 0.001 and **p < 0.01.

In Task 1, we selected a subset of rats (16 of 23) that underwent extinction training and avoidance testing, to assess neural activation using the activity marker c-Fos (Figures 3A,B). The subset of rats expressed a wide range of avoidance values at test, in order to assess co-variance with c-Fos expression. We observed significant positive correlations between avoidance at test and c-Fos density in PL (*r* = 0.79, *p* < 0.001), VS (*r* = 0.86, *p* < 0.001) and BA (*r* = 0.67, *P* = 0.0046; Figure 3C), consistent with findings from pharmacological inactivation studies of platform-mediated avoidance (Bravo-Rivera et al., 2014b).

Furthermore, avoidance at test correlated negatively with c-Fos density in IL (*r* = −0.74, *p* < 0.001, Figure 3C), consistent with impaired retrieval of extinction following inactivation of IL (prior to extinction) in this task (Bravo-Rivera et al., 2014b). Moreover, c-Fos density in IL correlated negatively with c-Fos density in BA (*r* = −0.65, *p* = 0.007; Figure 3E), with rats separating into two clusters: (1) those with high avoidance (with increased and decreased activity in BA and IL, respectively); and (2) those with low avoidance (with decreased and increased activity in BA and IL, respectively). Given the established roles of IL in fear extinction (Fontanez-Nuin et al., 2011; Sierra-Mercado et al., 2011; Do-Monte et al., 2015a) and BA in fear expression (Anglada-Figueroa and Quirk, 2005; Herry et al., 2008), persistent avoidance in Task 1 appears to correlate with poor extinction of fear.

### c-Fos Expression at Test in Task 2

We next characterized activity patterns during persistent avoidance in Task 2 by selecting a subgroup of rats (12 out of 22) showing a wide range of avoidance values at test. Similar to Task 1, we observed positive correlations between avoidance expression and c-Fos density in PL (*r* = 0.85, *p* < 0.001) and VS (*r* = 0.77, *p* = 0.0043; Figure 3D). Unlike Task 1, however, avoidance expression was not correlated with c-Fos density in IL (*r* = 0.41, *p* = 0.19) or BA (*r* = −0.17, *p* = 0.60; Figure 3D). In fact, all rats showed high activity levels in IL, and most rats showed low activity levels in BA, consistent with successful fear extinction. Furthermore, there was no clustering of rats into behavioral subgroups in the BA vs. IL plot (Figure 3F): rats with high IL and low BA activity exhibited both high and low avoidance.

## Discussion

In this study, we sought to characterize persistent avoidance using a platform-mediated avoidance task. When extinction tones were delivered in the presence of the platform (Task 1), persistent avoidance resembled deficient extinction of tone-shock associations, as evidenced by high freezing and c-Fos expression patterns. However, when extinction tones were delivered in the absence of the platform (Task 2), persistent avoidance still occurred despite successful extinction of tone-shock associations.

In Pavlovian fear extinction, there is considerable variability in the subsequent retrieval of extinction (Bush et al., 2007; Sotres-Bayon et al., 2008), and such failure is associated with a pattern of c-Fos similar to what we observed in Task 1 (Herry and Mons, 2004; Berretta et al., 2005; Knapska and Maren, 2009; Kim et al., 2010). Thus, persistent avoidance in Task 1 could simply reflect poor tone-shock extinction. However, correlations do not imply causality, and there was no correlation between freezing and avoidance at test in Task 1. The relationship between extinction of fear and avoidance was specifically addressed in Task 2, in which tone-shock associations were separately extinguished to criterion prior to the avoidance test. This manipulation reduced freezing at test (as expected) but did not reduce avoidance. Thus, persistent avoidance in Task 2 (and perhaps Task 1 as well) could be operating independently from fear extinction.

The only structures in both tasks that were positively correlated with avoidance were PL, VS, and OFC (see Tables 1, 2). PL and VS likely play a role in avoidance expression, given that inactivation of PL (Beck et al., 2014; Bravo-Rivera et al., 2014b) or VS (Bravo-Rivera et al., 2014b; Ramirez et al., 2015) impairs the expression of avoidance. PL may mediate avoidance through projections to VS (Bravo-Rivera et al., 2014a; Lee et al., 2014). In contrast, activity in BA and IL were only correlated with avoidance in Task 1, suggesting that these areas do not mediate avoidance expression *per se*. IL mediates extinction of avoidance (Bravo-Rivera et al., 2014b), as well as extinction of conditioned fear (Burgos-Robles et al., 2007; Laurent and Westbrook, 2009; Amir et al., 2011; Fontanez-Nuin et al., 2011; Sierra-Mercado et al., 2011; Do-Monte et al., 2015a). Our observation that IL activity was deficient in persistent rats is likely due to a reduction in IL activity during extinction training, rather than at test (Do-Monte et al., 2015a). BA has been reported to mediate avoidance (Lázaro-Muñoz et al., 2010; Ramirez et al., 2015) and inactivation of BA reduces expression of platform-mediated avoidance (Bravo-Rivera et al., 2014b). However, the lack of correlation in Task 2 instead suggests that BA signals the tone-shock association, which is reduced in Task 2 by extinction to criterion in the absence of the platform. In fact, at test, the majority of rats in Task 2 (9 of 12) showed the pattern of c-Fos consistent with successful tone-shock extinction (low BA, high IL, Figure 3F; Knapska and Maren, 2009), yet were mixed in their expression of avoidance. Activity in the CeM and the PVT correlated with avoidance in Task 1, as shown in Table 1. Both CeM (LeDoux et al., 1988; Goosens and Maren, 2001) and PVT (Do-Monte et al., 2015b; Penzo et al., 2015) are key mediators of conditioned fear. Moreover, a recent study showed that activity in CeM correlated with shuttle avoidance (Martinez et al., 2013).

**Table 1:** **Correlations between avoidance expression and c-Fos density in Task 1**.

Structure	*R*	*P* value
PL	0.79*	<0.001
IL	−0.74*	<0.001
VS	0.86*	<0.001
BA	0.67*	0.0046
CeM	0.75*	<0.001
CeL	−0.48	0.063
PVT	0.57	0.023
OFC	0.79*	<0.001

Legend: PL, prelimbic cortex; IL, infralimbic cortex; VS, ventral striatum; BA, basal amygdala; CeM, centromedial nucleus of the amygdala; CeL, centrolateral nucleus of the amygdala; PVT, paraventricular nucleus of the thalamus; OFC, orbitofrontal cortex. *Depicts significanct correlation after Bonferroni correction.

**Table 2:** **Correlations between avoidance expression and c-Fos density in Task 2**.

Structure	*R*	*P* value
PL	0.85*	<0.001
IL	0.40	0.19
VS	0.77*	0.0043
BA	−0.16	0.19
CeM	0.55	0.043
CeL	−0.34	0.23
PVT	0.50	0.072
OFC	0.87*	<0.001

Legend: PL, prelimbic cortex; IL, infralimbic cortex; VS, ventral striatum; BA, basal amygdala; CeM, centromedial nucleus of the amygdala; CeL, centrolateral nucleus of the amygdala; PVT, paraventricular nucleus of the thalamus; OFC, orbitofrontal cortex. *Depicts significanct correlation after Bonferroni correction.

If not a deficiency in tone-shock extinction, what factors might be generating persistent avoidance in Task 2? (1) Returning the platform at test could trigger renewal of fear, if rats associated the platform with the occurrence of shock (Bouton and King, 1983). This is unlikely however, because all rats showed low freezing at test. (2) Avoidance may have been driven by non-fearful motivations such as habit learning (Atallah et al., 2007; Balleine and O’Doherty, 2010), or increased value estimation (Berridge et al., 2009). Interestingly, the latter is dependent on the OFC, which was positively correlated with avoidance in both tasks. However, our data do not dissociate possible roles of OFC in avoidance prior to extinction vs. after extinction. Other studies have shown that avoidance could be extinguished independently from fear (Lolordo and Rescorla, 1966; Riccio and Silvestri, 1973), suggesting that fear and avoidance circuits are dissociable.

Avoidance is a core symptom of anxiety disorders (Kashdan et al., 2006) and a prominent feature of Post Traumatic Stress Disorder (PTSD; Friedman et al., 2011; American-Psychiatric-Association, 2013). Prolonged exposure therapy is based on fear extinction and it is the standard of care for PTSD (Davis et al., 2006; Foa, 2006; Kearns et al., 2012). However, extinction-based therapies often do not reduce avoidance behaviors (Sripada et al., 2013). Therefore, avoidance can occur independent of fear, suggesting that therapies that reduce fear may not be useful in reducing persistent avoidance behaviors.

Human neuroimaging studies of active avoidance are beginning to emerge, and recent findings implicate a prefrontal-cingulate-striatal circuit (Delgado et al., 2009; Aupperle et al., 2015), consistent with the involvement of PL and VS in active avoidance (Bravo-Rivera et al., 2014b; Lee et al., 2014). Distinguishing extinction of fear from extinction of avoidance could help identify substrates of persistent avoidance in humans, and may help guide treatments for avoidance-related disorders such as PTSD.

## Conflict of Interest Statement

The authors declare that the research was conducted in the absence of any commercial or financial relationships that could be construed as a potential conflict of interest.
